# Resonance assignments for latherin, a natural surfactant protein from horse sweat

**DOI:** 10.1007/s12104-013-9485-3

**Published:** 2013-05-26

**Authors:** Steven J. Vance, Rhona E. McDonald, Alan Cooper, Malcolm W. Kennedy, Brian O. Smith

**Affiliations:** 1 School of Chemistry, College of Science and Engineering, University of Glasgow, Glasgow, G12 8QQ UK; 2 Institute of Molecular, Cell and Systems Biology, College of Medical, Veterinary and Life Sciences, University of Glasgow, Glasgow, G12 8QQ UK; 3 Institute of Biodiversity, Animal Health and Comparative Medicine, College of Medical, Veterinary and Life Sciences, University of Glasgow, Glasgow, G12 8QQ UK; 4 Present Address: Department of Biochemistry, School of Biological Sciences, University of Cambridge, Cambridge, CB2 1GA UK; 5 Present Address: Life Sciences Lead, Strategic Trade, UK Trade and Investment, 1 Victoria Street, London, SW1H 0ET UK

**Keywords:** Latherin, Surfactant protein, Horse, Sweat, Saliva, Allergen, NMR

## Abstract

Latherin is an intrinsically surfactant protein of ~23 kDa found in the sweat and saliva of horses. Its function is probably to enhance the translocation of sweat water from the skin to the surface of the pelt for evaporative cooling. Its role in saliva may be to enhance the wetting, softening and maceration of the dry, fibrous food for which equines are adapted. Latherin is unusual in its relatively high content of aliphatic amino acids (~25 % leucines) that might contribute to its surfactant properties. Latherin is related to the palate, lung, and nasal epithelium carcinoma-associated proteins (PLUNCs) of mammals, at least one of which is now known to exhibit similar surfactant activity to latherin. No structures of any PLUNC protein are currently available. ^15^N,^13^C-labelled recombinant latherin was produced in *Escherichia coli*, and essentially all of the resonances were assigned despite the signal overlap due to the preponderance of leucines. The most notable exceptions include a number of residues located in an apparently dynamic loop region between residues 145 and 154. The assignments have been deposited with BMRB accession number 19067.

## Biological context

Several types of unrelated proteins appear to exhibit intrinsic surfactant activity as their primary function, which, for the moment, appears to be the case for latherin. Latherin is one of the most abundant proteins in the sweat of horses, it is also found in horse saliva, and is a known allergen to some humans (McDonald et al. 2009). Latherin’s function is believed to be to wet the hydrophobic hairs in order to enhance the rate of translocation of sweat water to the surface of the pelt for evaporative cooling (McDonald et al. 2009). The hydrophobins, a family of surface active proteins produced by filamentous fungi, and RSN-2, a surfactant protein present in the foam nests of certain species of frogs, have been investigated at the protein structure level (Linder 2009; Cooper et al. 2005; Fleming et al. 2009). Latherin exhibits no amino acid sequence similarities to either of these proteins, but is instead a member of the palate, lung, and nasal epithelium carcinoma-associated family of proteins (PLUNCs) found in mammals (McDonald et al. 2009). Latherin and PLUNCs are, in turn, related to the larger, two-domain bactericidal/permeability-increasing protein (BPI), cholesteryl ester-transfer protein (CETP) and lipopolysaccharide-binding protein (LBP). Although the functions of individual PLUNCs have not been confirmed, they are postulated to have some role within the innate immune response (Bingle and Craven 2002). The structures of BPI and CETP are available, but no structure for any member of the PLUNC family has thus far been reported.

Latherin’s amino acid sequence is unusually rich in aliphatic residues, in particular leucine, which contributes almost 25 % of the residues present, compared to the SwissProt average for all proteins within that database of 9.67 % (McDonald et al. 2009). This abundance of leucines is also a feature of one of the PLUNCs from humans that, like latherin, exhibits strong surfactant activity (Gakhar et al. 2010). Latherin, therefore, not only presents an opportunity to investigate the relationship between structure and function of a unique surfactant protein of mammals, but potentially also to understand the structure and function of the PLUNCs as a whole, for which there is currently little or no structural and direct functional information.

## Methods and experiments

A synthetic latherin (sLath) gene based upon the previously described, cDNA encoding latherin (GenBank AF491288; UniProt/Swiss-Prot P82615), excluding the presumptive secretory leader/signal peptide, optimised for expression in *Escherichia coli,* was purchased from GeneArt. The sLath gene was then directionally inserted into the NcoI, BamHI sites of the pET32a expression vector (Novagen) allowing for the production of recombinant latherin extended by two extra amino acids, AM (single letter amino acid code) at the N-terminus of the wild-type sequence. The ‘sLath/pET32’ plasmid was transformed into Tuner (DE3) cells (Novagen). Expression was carried out in Luria–Bertani broth for non-labeled samples or M9 minimal media (Sambrook et al. 1989) containing the relevant isotope(s) for the production of single (^15^N only) or double (^15^N, ^13^C) labeled samples. The protein was purified to near homogeneity as estimated from SDS-PAGE electrophoresis, as described previously (McDonald et al. 2009).

For the purpose of NMR, protein was concentrated to approximately 600 μM in 50 mM NaCl, 20 mM sodium phosphate, 1 mM sodium azide, pH 7.5. D_2_O was added to a final concentration of 5 % (v/v). All experiments were performed at 310 °K using a Bruker AVANCE 600 MHz spectrometer equipped with 5 mm triple-resonance probes and pulsed-field gradients. The WATERGATE tailored selective excitation sequence was typically used for water suppression (Piotto et al. 1992). Proton chemical shifts were referenced relative to the H_2_O offset frequency and heteronuclear chemical shifts calculated from the proton reference according to the method of Wishart et al. (1995). NMR spectra were processed using AZARA (Wayne Boucher, Department of Biochemistry, University of Cambridge, http://www.bio.cam.ac.uk/azara) and assigned using CCPNmr analysis (Vranken et al. 2005). Maximum entropy reconstruction (Laue et al. 1986) was used to enhance resolution of the indirect dimensions of three-dimensional experiments.

**Fig. 1:**
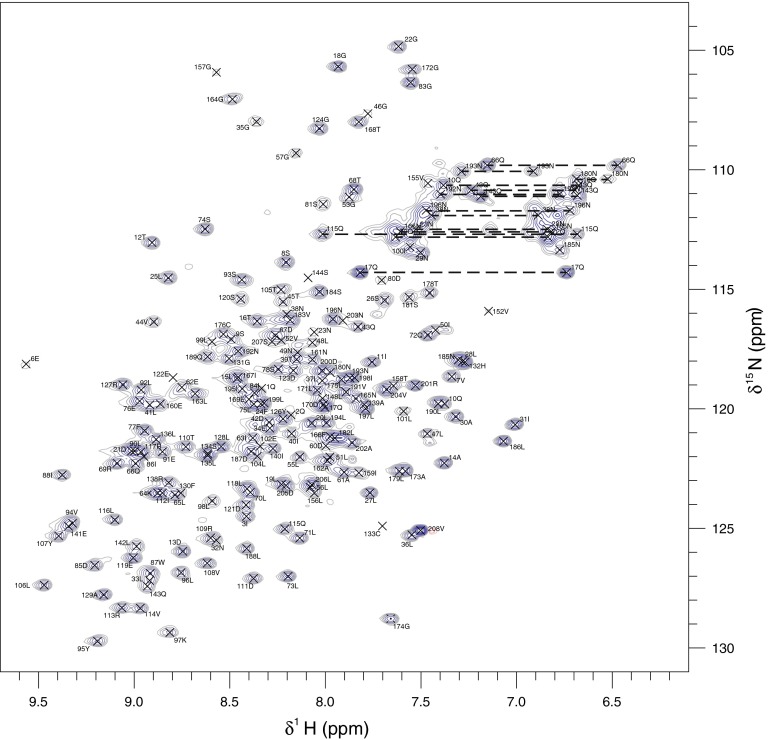
The ^15^N HSQC spectrum of latherin at 310 K. The residue specific assignments are indicated and the crosspeaks assigned to sidechain NH2 groups are linked by *horizontal dashed lines*

**Fig. 2:**
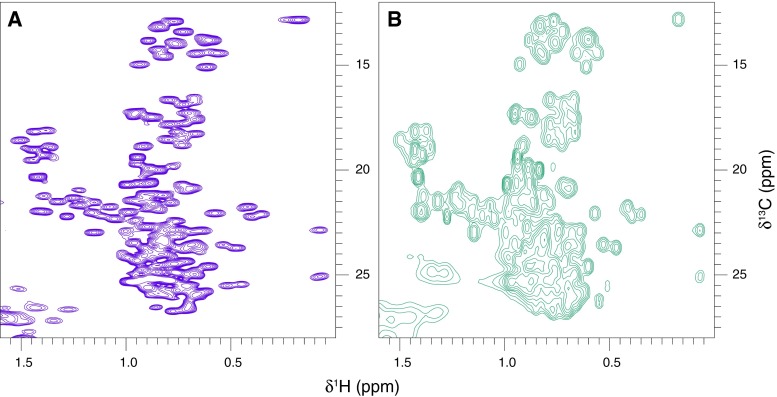
The methyl region of the ^13^C, ^1^H correlation spectra of latherin illustrating the increased resolution of the leucine methyl crosspeaks possible with **a** the me-HCCH-TOCSY experiment as compared to **b** the conventional ^13^C-HSQC

Sequence-specific resonance assignment of the latherin backbone was accomplished with the aid of 2D ^15^N-HSQC (see Fig. 1), 3D HNCACB, 3D CBCA(CO)NH (Muhandiram and Kay 1994), 3D HNCO (Kay et al. 1994), 3D HNCACO, 3D HBHA(CBCA)NH (Wang et al. 1994) and HBHA(CBCACO)NH spectra. The majority of aliphatic sidechain carbon and proton resonances were located by navigating from the backbone data using 2D ^13^C-HSQC, 3D (H)C(CO)NH-TOCSY, 3D and 3D H(C)(CO)NH-TOCSY spectra (Grzesiek and Bax 1992). The high number of overlapping leucine sidechain resonances were assigned using 3D methyl-selective experiments (Uhrin et al. 2000) modified for the removal of CH_2_ resonances from the methyl proton-carbon planes (see Fig. 2). Remaining aliphatic resonances were identified using 3D ^13^C-edited [^1^H, ^1^H]-NOESY spectra. A proportion of aromatic sidechain ^13^C/^1^H signals (histidine Hδ1, tryptophan Hδ1, tyrosine Hδ,ε and phenylalanine Hδ,ε) were assigned using 2D HBCBCGCDHD and 2D HBCBCGCDCEHE spectra (Yamazaki et al. 1993) and the remainder were identified from the ^13^C-edited [^1^H, ^1^H]-NOESY spectrum.

## Extent of assignments and data deposition

All latherin polypeptide backbone resonances were assigned, with the exception of the N-terminal residues A(−2), M(−1), A(0); two isolated residues S59, K82; and a number of residues located on a dynamic loop region (G145, N146, S149, L150, N153, A154). A total of 93.51 % of backbone residues were identified, while assignment of non-labile amino acid sidechain protons is 94.23 % complete. The majority of the missing assignments are those of the residues within the 145–154 residue dynamic loop region. Despite their high relative abundance within the protein, the experiments nevertheless allowed full assignment of all leucine residues. A few resonances displayed chemical shifts outwith the known distribution of shifts. Sidechain protons in residues (85D, 113R and 180 N) all displayed the effects of ring current shift due to their close proximity to aromatic residues. 135L C_γ_ has an atypical chemical shift of 31.09 ppm. This residue is buried within the hydrophobic core of the protein surrounded by other aliphatic residues, and analysis of its stereochemical properties in the calculated structure indicated φ, Ψ, χ_1_ and χ_2_ bond angles in favourable regions. The atypical chemical shift in 135L therefore remains to be explained.

The ^1^H, ^13^C and ^15^N chemical shift assignments have been deposited with the BioMagResBank database (http://www.bmrb.wisc.edu), accession number 19067.
